# Severe Acute Transverse Myelitis With a Rapid Progression in an Infant

**DOI:** 10.7759/cureus.72558

**Published:** 2024-10-28

**Authors:** Cátia Martins, Joana Verdelho Andrade, Ema Grilo, Filipe Palavra, Rita Moinho

**Affiliations:** 1 Pediatric Intensive Care Unit, Hospital Pediátrico, Unidade Local de Saúde de Coimbra, Coimbra, PRT; 2 Pediatric Palliative Care Team, Hospital Pediátrico, Unidade Local de Saúde de Coimbra, Coimbra, PRT; 3 Neuropediatric Unit, Centre for Child Development, Hospital Pediátrico, Unidade Local de Saúde de Coimbra, Coimbra, PRT; 4 Laboratory of Pharmacology and Experimental Therapeutics, Coimbra Institute for Clinical and Biomedical Research (iCBR), Faculty of Medicine, University of Coimbra, Coimbra, PRT

**Keywords:** case report, child, quadriplegia, transverse myelitis, urinary retention

## Abstract

Acute transverse myelitis (ATM) is a rare inflammatory disorder of the spinal cord, often triggered by infections, systemic diseases, or occurring without a known cause (idiopathic). It is a diagnosis of exclusion, characterized by motor, sensory, and autonomic dysfunction, with spinal cord inflammation confirmed through cerebrospinal fluid analysis or MRI. While most children experience a favorable recovery, severe cases can lead to lasting neurological deficits.

This report presents the case of a 10-month-old male who presented with severe para-infectious ATM, likely triggered by *Campylobacter* *spp.* enteritis, confirmed through fecal polymerase chain reaction. The infant developed rapid-onset motor deficits, progressing to flaccid tetraparesis and respiratory failure, needing prolonged invasive mechanical ventilation and pediatric intensive care unit admission. MRI revealed extensive spinal cord involvement from C1 to D4-D5. Despite corticosteroid therapy and immunoglobulin infusion, therapeutic plasma exchange was also performed. Recovery was slow, and the patient achieved ventilatory autonomy but continued to experience significant neurological deficits, including spastic tetraparesis, and a neurogenic bladder requiring intermittent catheterization. After 73 days, the patient was discharged but required ongoing intensive rehabilitation.

This case underscores a critical episode of ATM marked by multiple adverse prognostic factors. It emphasizes the necessity of comprehensive treatment approaches, including life support, efforts to reverse the inflammatory process in the spinal cord, and extensive rehabilitation. Furthermore, it highlights the importance of collaborative healthcare teams in striving for optimal patient outcomes.

## Introduction

Acute transverse myelitis (ATM) is an inflammatory disease of the spinal cord, often associated with infectious, vascular, systemic inflammatory, or neoplastic conditions [1]. However, in the majority of cases, no specific cause is identified, suggesting an immune-mediated etiology, classifying the condition as idiopathic [2]. The estimated incidence of ATM is one to three cases per million children per year, with a slight male predominance (0.85:1 ratio) [1,3]. The median age of affected children is approximately 10 years, and a bimodal age distribution has been noted, with a peak incidence occurring below 3 years and between 5 and 17 years of age [1,3-5].

ATM is a diagnosis of exclusion, with diagnostic criteria established by the Transverse Myelitis Consortium Working Group [3]. It presents with bilateral motor, sensory, and autonomic dysfunction attributable to spinal cord involvement, with a clearly defined sensory level. Inflammation of the spinal cord is confirmed by cerebrospinal fluid (CSF) pleocytosis, elevated CSF immunoglobulin G (IgG) index, or gadolinium enhancement on magnetic resonance imaging (MRI), and central nervous system infection must be ruled out. Symptom progression typically reaches its nadir within 4 hours and 21 days of onset [3,6].

Children usually have a favorable motor prognosis, with complete recovery occurring in up to 56% of cases, and a mean time to regaining the ability to walk of 56 days [7].

We present the case of a 10-month-old infant diagnosed with severe ATM, likely of para-infectious etiology.

## Case presentation

**Figure 1:**
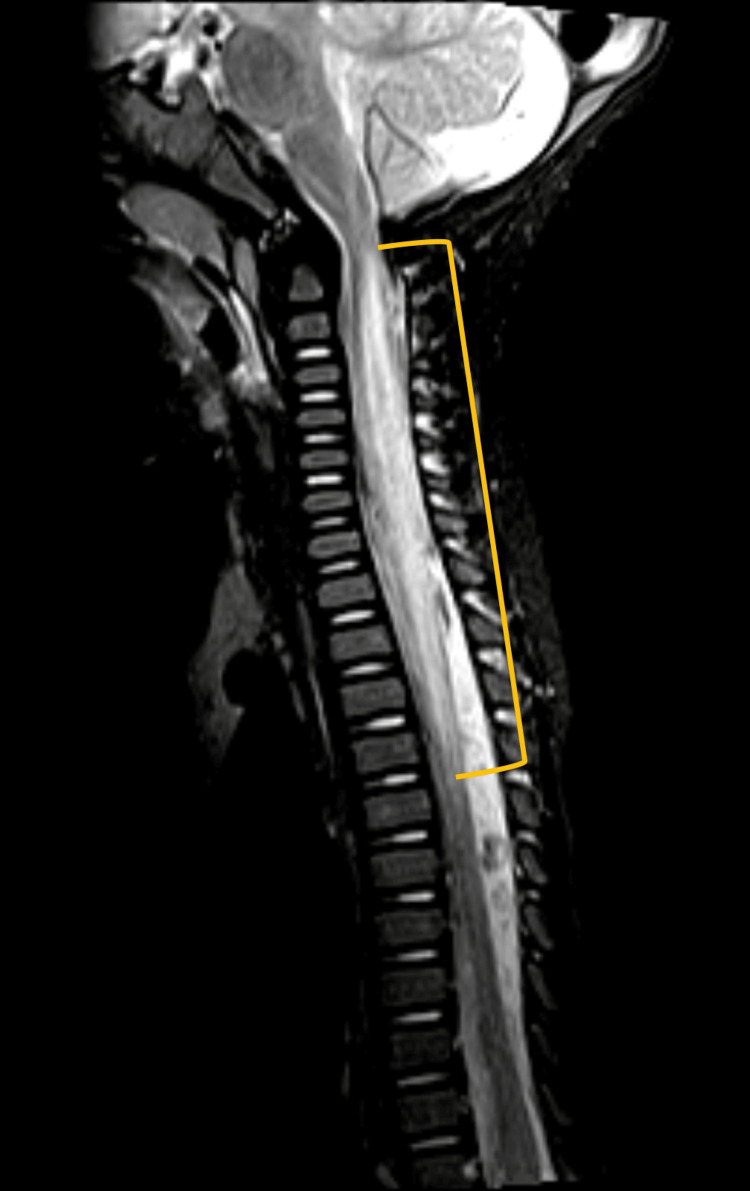
Magnetic resonance imaging of the spinal cord (T2/STIR sequence, sagittal plan) showing longitudinal extensive myelitis from the lower plane of C1 to D4-D5 (marked in yellow).

A previously healthy 10-month-old male infant presented to the emergency department with a sudden onset of motor deficits, initially affecting the upper limbs and progressively involving the lower limbs within hours. Six days prior to presentation, the infant had a one-day history of fever and diarrhea. On examination, he was irritable, had a weak cry, and exhibited flaccid tetraparesis, most pronounced in the upper limbs. Myotatic reflexes were present and symmetrical in the upper limbs, with brisk reflexes in the lower limbs. There was a rapidly exhaustible Achilles clonus on the left side, and plantar cutaneous reflexes were bilaterally indifferent. Ocular mobility was intact, with no tongue fasciculations or dysphagia. The patient’s condition worsened in the first hours following admission, developing weak cough, respiratory secretions accumulation, and respiratory distress, though without hypoxemia or hypercapnia. Despite these respiratory challenges, he remained hemodynamically stable. Due to worsening respiratory function, he was admitted to the pediatric intensive care unit (PICU). Cranial and neuroaxis MRI revealed a longitudinally extensive myelitis from C1 to D4-D5, with transverse and diffuse spinal cord involvement. There was notable medullary expansion, especially between C3 and D1, with marked long T2/STIR hypersignal (Figure 1), faint T1 hypointensity, and no evident gadolinium enhancement. Lumbar puncture showed mildly elevated protein levels (50 mg/dL), normal glucose, and cytological analysis revealed pleocytosis (35 leukocytes/mm^3^), with negative bacterial and viral polymerase chain reaction (PCR) results. Additionally, he had elevated serum IgG and a decreased CSF IgG index. No antibodies or oligoclonal bands were detected in the CSF (including anti-aquaporin 4 and anti-MOG), and all serum antibody tests returned negative.

On the first day of hospitalization, the patient's respiratory function deteriorated, with ineffective coughing and accumulation of secretions, necessitating intubation. He received intravenous immunoglobulin (IVIG) therapy (2 g/kg) and seven daily pulses of methylprednisolone (30 mg/kg/day) until day 8, followed by a gradually tapering course of systemic corticosteroids, reaching a maintenance dose of 1.2 mg/kg/day. On the 9th day, due to minimal improvement, persistent flaccid tetraparesis, and ventilator dependency, plasmapheresis was initiated. He underwent seven cycles of therapeutic plasma exchange (TPE) on alternate days, with the treatment course completed by day 22. Each session involved a 1.1-1.3 plasma volume exchange. The first cycle was performed using albumin alone, which resulted in several adverse effects, including metabolic acidosis requiring correction and various electrolyte imbalances. For the subsequent cycles, a combination of 50% albumin and 50% plasma was used, leading to a significant reduction in these side effects. However, following the procedures, he experienced transient hemodynamic instability, necessitating fluid boluses and short-term vasopressor support.

During his PICU stay, PCR testing detected enterovirus in respiratory secretions (negative for enterovirus D68) and *Campylobacter* spp. in stools (despite negative stool cultures), leading to a five-day course of azithromycin. Clinical improvement was slow. By day 23, neurological evaluation revealed persistent flaccid tetraparesis predominantly affecting the upper limbs with slight contraction of the right upper limb and mild flexion of the right thigh in response to painful stimuli. Patellar reflexes were elicitable and symmetrical, but other reflexes remained absent. The plantar cutaneous reflex showed bilateral extension.

Mechanical ventilation was required until the 23rd day, after which non-invasive ventilation (NIV) was initiated, with progressively longer tolerated pauses. Daily respiratory physiotherapy and cough assist were crucial for ventilatory recovery. Bladder catheterization was required during the PICU stay, while bowel movements remained spontaneous and regular.

On day 29, the patient was discharged from the PICU and continued under the pediatric palliative care team due to significant physical impairments, requiring several medical devices, and uncertainty about recovery. An intensive rehabilitation plan was followed, with slow clinical progress. NIV was needed until day 50, and the nasogastric tube was used for feeding until day 40, after which the patient was fed exclusively orally. He was discharged from the hospital after a total of 73 days, continuing on corticosteroid therapy (1.2 mg/kg/day) with a gradual taper over six months, alongside regular clinical monitoring. The family received comprehensive training in home care, focusing on respiratory support while also ensuring regular physiotherapy and speech therapy aligned with the rehabilitation team's plan. At 20 months of age, the patient is fed orally without dysphagia or choking, but continues to require cough assist twice daily for secretion management, although NIV is no longer needed. He has developed a hyperactive neurogenic bladder, requiring intermittent catheterization. He is still on physiotherapy, occupational, and speech therapy two to three times per week, showing gradual improvements, including the ability to articulate a few words. Neurologically, he has intercostal muscle weakness and abdominal breathing, with no other cranial nerve abnormalities. He has spastic tetraparesis with a muscle strength of 3/5 in the upper limbs and 2/5 in the lower limbs, lacks effective trunk control, and, when standing, relies on extensor responses and upper limb support to maintain posture.

## Discussion

ATM is a non-compressive inflammatory myelopathy, with around 20% of cases occurring in children and adolescents under 18 years of age [5]. It can present as part of an acquired recurrent demyelinating syndrome, such as multiple sclerosis, acute disseminated encephalomyelitis, and neuromyelitis optica spectrum disorders [2]. The clinical presentation depends on the level of spinal cord involvement. Differential diagnosis should consider both intrinsic and extrinsic spinal cord pathologies [1]. Acute flaccid myelitis (AFM) is an important differential diagnosis to consider in ATM cases, mainly due to the different treatment strategies, because in AFM the use of steroids can worsen the clinical outcome [8]. AFM typically presents with rapidly progressive flaccid limb weakness caused by damage to the anterior horn cells in the spinal cord. The diagnostic criteria include acute onset of limb weakness with prodromal fever or illness; weakness involving one or more limbs, neck, face, or cranial nerves; decreased muscle tone in at least one weak limb; decreased or absent deep tendon reflexes in at least one weak limb; and MRI with spinal cord lesion with predominant grey matter involvement, with or without nerve root enhancement and CSF pleocytosis [9]. In this disease, upper limb weakness is often more pronounced than lower limb involvement [8]. The condition is associated with viral infections, likely through the invasion of anterior horn cells. Poliovirus was historically a common cause, but following successful vaccination campaigns, non-polio enteroviruses, particularly enterovirus D68 and A71, have been increasingly implicated [8,9]. In this case, the predominance of lower limb involvement with hyperreflexia, bladder dysfunction, and diffuse spinal cord lesions on MRI strongly favor a diagnosis of ATM over AFM. Although enterovirus was detected in the patient’s respiratory secretions via PCR, enterovirus D68 was not identified.

Children with ATM generally have a better prognosis than adults, with approximately half achieving full recovery by two years [5]. This case is notable for its severity and rapid progression to nadir, requiring intubation on the first day of hospitalization, which is a finding consistent with other reports [1,5]. Poor prognostic factors in this patient included age under three years, rapid progression to nadir in less than 24 hours, and early bladder dysfunction [1,5]. Other predictors of poor outcome, such as the absence of CSF pleocytosis, gadolinium enhancement on MRI, and lack of cervical or cervicothoracic involvement, were not present in this case [1,5].

In line with other reports, which indicate that infections are identified in 47-60% of cases, an infectious etiology was also identified in this patient [5,6]. Campylobacter infection is a recognized but uncommon cause of para-infectious ATM, as supported by several case reports. Although the pathophysiology is not fully understood, it is believed that the infection triggers an inflammatory process in the central nervous system [10,11]. In this patient, recent enteritis caused by *Campylobacter *spp. was confirmed through fecal PCR, making para-infectious ATM the most likely etiology after exclusion of other potential causes.

The MRI findings in this case revealed an extensive spinal cord lesion, from C1 to D4-D5, which is more diffuse than typically reported. Most cases in the literature describe more localized lesions, with 50% involving the cervical spine and 40% the thoracic spine [5].

This infant required prolonged PICU care and multiple therapies, reflecting the severe nature of his disease. Currently, the optimal acute treatment for ATM in children remains unclear and is primarily based on case series and expert opinion, as randomized controlled trials are lacking [12]. High-dose corticosteroids, such as 30 mg/kg/day of methylprednisolone given to this patient, are generally recommended in the acute phase. Early corticosteroid therapy, administered for three to seven days, appears to improve short and long-term outcomes. This approach is associated with a higher proportion of patients achieving complete recovery, faster regaining of gait function, and reduced severity of sequelae [13,14]. Following initial treatment, low-dose corticosteroid therapy (1 mg/kg/day) should be maintained, with a gradual taper over three to four weeks [14], as it was done in this case. Due to the lack of significant improvement with corticosteroids and persistent significant motor and respiratory deficits, seven cycles of TPE were performed. Various studies support this technique, either concomitant with corticosteroid therapy or subsequently, if there is no response [2]. TPE is a safe and effective treatment, with protocols typically including five to seven sessions on alternate days, exchanging 1.1 to 1.5 plasma volumes [12,15,16]. The American Academy of Neurology's guidelines recommend considering TPE in patients who fail to respond to corticosteroids [15]. In this case, clinical improvement became more consistent after TPE, suggesting it was the most effective therapy, although the benefit of the combined effects of other treatments cannot be ruled out. IVIG therapy, at a dose of 2 g/kg over two to five days, was also administered in our patient, though its role in ATM treatment is less clearly defined. Despite limited evidence, IVIG is often included in treatment regimens for fulminant disease [2].

Although the prognosis in pediatric ATM is generally more favorable than in adults, some children experience severe sequelae, and, in rare instances, mortality occurs, primarily due to respiratory failure or extensive cervical spinal cord lesions [5]. Mechanical ventilation appears to be associated with a reduced likelihood of achieving ambulatory function, with only 39% of patients who were ventilated able to walk, compared to 59% of patients who did not require ventilation [5]. The most common sequelae are sensory disturbances and bladder dysfunction, with the latter being the most common long-term neurological deficit [5]. Up to 25% of children with ATM are unable to walk again, and 10-20% do not recover bladder function [2]. In this case, despite slow progress over 10 months of follow-up, with the child presenting with spastic tetraparesis, an inability to walk, and the need for intermittent bladder catheterization, there are positive aspects to highlight, such as the maintenance of respiratory stability, the recovery of oral feeding ability, and the recent ability to speak a few words. However, the long-term neurological outcome in this patient is still uncertain, as it remains too early to assess the full extent of recovery.

## Conclusions

This case highlights a severe episode of ATM marked by multiple poor prognostic factors and the need for various treatments, including both life support and reversal of the inflammatory process in the spinal cord. Although the clinical improvement should be attributed to the combination of all treatments provided, TPE seems to have played an important role in the functional recovery of this child, particularly in regaining ventilatory autonomy. This case also underscores the importance of a multidisciplinary approach, with coordinated care among different healthcare teams playing a vital role in supporting both the child and their family through each phase of the disease.
